# Development of a 2-Channel Embedded Infrared Fiber-Optic Temperature Sensor Using Silver Halide Optical Fibers

**DOI:** 10.3390/s111009549

**Published:** 2011-10-11

**Authors:** Wook Jae Yoo, Kyoung Won Jang, Jeong Ki Seo, Jinsoo Moon, Ki-Tek Han, Jang-Yeon Park, Byung Gi Park, Bongsoo Lee

**Affiliations:** 1 School of Biomedical Engineering, College of Biomedical & Health Science, Konkuk University, 322 Danwol-dong, Chungju-si, Chungcheongbuk-do, 380-701, Korea; E-Mails: wonzip@naver.com (W.J.Y.); kko988@hotmail.com (K.W.J.); halcyonost@naver.com (J.K.S.); mjs092082@nate.com (J.M.); nikev2@nate.com (K.-T.H.); jyparu@kku.ac.kr (J.-Y.P.); 2 Department of Energy & Environment Engineering, College of Engineering, Soonchunhyang University, 646 Eupnae-ri, Shinchang-myeon, Asan-si, Chungcheongnam-do, 336-745, Korea; E-Mail: byunggi@sch.ac.kr

**Keywords:** embedded sensor, fiber-optic sensor, infrared, silver halide optical fiber, thermometry

## Abstract

A 2-channel embedded infrared fiber-optic temperature sensor was fabricated using two identical silver halide optical fibers for accurate thermometry without complicated calibration processes. In this study, we measured the output voltages of signal and reference probes according to temperature variation over a temperature range from 25 to 225 °C. To decide the temperature of the water, the difference between the amounts of infrared radiation emitted from the two temperature sensing probes was measured. The response time and the reproducibility of the fiber-optic temperature sensor were also obtained. Thermometry with the proposed sensor is immune to changes if parameters such as offset voltage, ambient temperature, and emissivity of any warm object. In particular, the temperature sensing probe with silver halide optical fibers can withstand a high temperature/pressure and water-chemistry environment. It is expected that the proposed sensor can be further developed to accurately monitor temperature in harsh environments.

## 1. Introduction

Thermocouples and resistance temperature detectors (RTDs) are widely used to measure temperature in industrial settings. However, there are difficulties in accurately measuring temperature in harsh environments using existing electrical sensors due to contamination or corrosion of the sensing probe and high electromagnetic interference (EMI) or radiofrequency interference (RFI).

Alternatively, optical fiber-based sensors may be used to measure physical properties including temperature. These sensors offer many advantages over conventional electrical sensors, including small size, good flexibility, remote operation, immunity to EMI or RFI, and resistance to harsh environments [1–3]. A number of optical thermometry methods using optical fibers have been developed, and it has been reported that fiber-optic sensors can measure temperature using infrared (IR) optical fibers [4–8], fiber Bragg gratings (FBGs) [9–11], or special materials that change some physical characteristic such as color, absorbance, and reflectance in accordance with temperature variation [12–15].

One of the simplest methods is IR radiometry using an IR optical fiber that can perform as a sensing material and an IR waveguide at the same time. Generally, it is possible to determine the temperature of any warm object by measuring the emitted blackbody radiation [16,17]. The operating principle of a radiometer using IR optical fiber is based on the relationship between the surface temperature of a heat source and the quality and the quantity of IR radiation [8,18].

**Figure 1.**
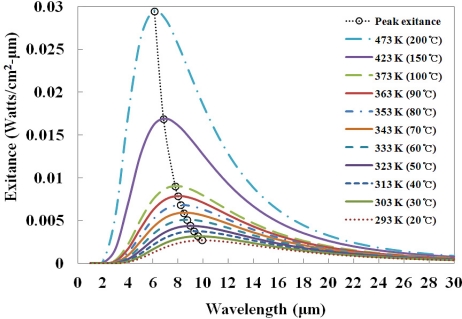
Relationship between the spectral exitance and the wavelength for various temperatures of a blackbody source.

According to blackbody radiation theory [19], total radiant exitance or emitted intensity (*I*) from a blackbody source at absolute temperature (*T*) is the integral over all wavelengths (*λ*):

(1) $$I=\int_{0}^{\infty }\frac{2\pi {\mathrm{hc}}^{2}}{\lambda^{5}[e^{\mathrm{hc}/\lambda \mathrm{kT}}-1]}d\lambda$$

where *h* is the Planck’s constant (*h* = 6.67 × 10^−34^ J·s), *c* is the speed of light in vacuum (*c* = 3 × 10^8^ m/s) and *k* is the Boltzmann constant, that is 1.38 × 10^−23^ [J/K]. Equation (1) can be interpreted as the area under the spectral exitance curves for a given temperature, as shown in Figure 1.

The intensity of emitted IR radiation depends on the temperature of the heat source, as delineated in Equation (2), called the Stefan-Boltzmann law [6,8,17,19]:

(2) $$I={ɛ\sigma }_{e}T^{4}[W/{\text{cm}}^{2}]$$

where *ɛ* is the emissivity of the heat source (0 ≤ *ɛ* ≤ 1) and *σ_e_* is the Stefan-Boltzmann constant for radiant exitance, that is, 5.67 × 10^−12^ W/(cm^2^·K^4^). The wavelength of peak exitance (*λ_max_*) is related to the temperature of the heat source by the Wien displacement law, as also shown in Figure 1. The decrease in the wavelength of peak exitance as the temperature increases can be quantified by Equation (3) [19]:

(3) $$\lambda_{\max}\;T=2897.8\;[\mu m\cdot K]$$

In this case, however, classical radiometers based on the blackbody radiation theory can only measure the surface temperature of a heat source in spite of their biggest advantages, such as non-contact measurement and excellent sensitivity to temperature difference. In addition, the output signal of the radiometer is influenced by the emissivity variation in accordance with the surface conditions of the heat source. The blackbody temperature that gives the same exitance as that of the measured intensity (*I*_meas_) is the radiation temperature. The blackbody temperature or the true temperature (*T*) can be calculated from the radiation temperature (*T_R_*) using Equation (2) if the measured heat source is a graybody whose emissivity is known. The corresponding mathematical expressions are as follows [19]:

(4) $$I_{\text{meas}}=ɛ\sigma T^{4}=\sigma T_{R}^{4}$$

(5) $$T={ɛ}^{-1/4}T_{R}$$

Normally, the radiation temperature is lower than the true temperature because the area under the spectral exitance curve is smaller. Therefore, it is necessary to develop a new concept for IR fiber-optic temperature sensors that can circumvent emissivity effects of the measured heat source and are independent of ambient temperature variation.

In this study, a 2-channel embedded IR fiber-optic temperature sensor was fabricated using two identical IR optical fibers for accurate thermometry without complicated calibration processes. Thermometry with the proposed fiber-optic sensor is immune to any changes of physical conditions and emissivity of a heat source. In addition, the proposed sensor can be directly embedded in a high temperature/pressure and water-chemistry environment, and can measure inner temperatures in real time. In order to accurately measure temperature, we measured the differences between the amounts of IR radiation emitted from two temperature sensing probes according to the temperature variation.

## 2. Materials and Experimental Setup

In fiber-optic thermometer applications, a chalcogenide optical fiber and a silver halide optical fiber are generally used as a representative IR waveguide [16,17]. A chalcogenide optical fiber, which can transmit light in the 1 to 6 μm range, has lower attenuation losses. In addition, a chalcogenide IR fiber based on binary glass arsenic trisulfide (As_2_S_3_) is non-hygroscopic and it shows excellent transmittance from 1.5 up to 6 μm, but on the other hand chalcogenide optical fibers are toxic and fragile, and their melting point is lower than 245 °C, therefore, a chalcogenide optical fiber is barely suitable for thermometry in high temperature/pressure and water-chemistry environments. In the case of a silver halide optical fiber based on silver bromide chloride (AgBrCl) polycrystalline material, temperature measurement is possible over a large temperature range because it is available for the transmission of mid-IR from 3 to 16 μm and its melting point is about 412 °C. Additionally, it has been reported that silver halide optical fiber is flexible, water-insoluble, and non-toxic [6].

**Figure 2.**
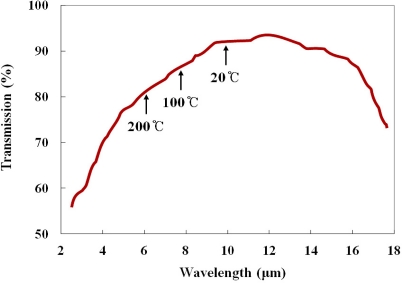
Transmission rates of a silver halide optical fiber.

As IR optical fiber, a silver halide optical fiber (JT Ingram, PIR 900/1000) was selected for this study. The outer diameter of this optical fiber is 1.0 mm, and the cladding thickness is 0.05 mm. The refractive indices of the core and the cladding are 2.15 and 2.13, respectively, and the numerical aperture (NA) is 0.25. This polycrystalline IR optical fiber is produced with pure AgCl:AgBr solid solution crystals in a core/clad structure, and the jacket is made of polyether ether ketone (PEEK). The silver halide optical fiber is transparent over a wide spectral range ranging from 4 to 18 μm, as shown in Figure 2, and the attenuation is less than 500 dB/km in a wavelength range from 9 to 14 μm [4]. This fiber is very flexible and durable over a temperature range from −200 to 250 °C, and its melting point is 415 °C.

**Figure 3.**
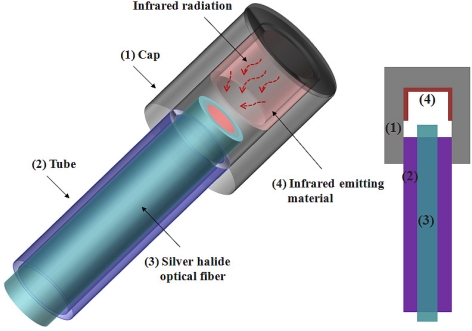
Structure of the temperature sensing probe.

Figure 3 illustrates the structure of the temperature sensing probe, which is composed of a cap (1), a tube (2), a silver halide optical fiber (3), and an IR emitting material (4). The cap and the tube are made of stainless steel to protect the silver halide optical fiber from the water-chemistry and harsh environment. For accurate thermometry and to circumvent any emissivity effects of the measured heat source the temperature sensing probes were divided into a signal probe (CH-1) and a reference probe (CH-2). The inner part of the cap of the signal probe was coated with high emissivity (*ɛ*_signal_ ≃ 1) black paint as an IR emitting material, while that of the reference probe was covered with a low emissivity polished stainless steel cap (*ɛ*_reference_ ≃ 0.1) [19]. The black paint (Motip Dupli, Dupli-color Supertherm), which is similar to a blackbody, has a rough surface and a high IR emissivity compared with the polished stainless steel. In addition, it is highly temperature-resistant up to 800 °C. The temperature of a heat source is determined by measuring the difference in intensity of the IR radiations emitted from the two kinds of materials in the caps. Therefore, the IR intensity difference (Δ*I*) is a function of the temperature of two probes, as delineated in Equation (6):

(6) $$\Delta I=I_{\text{signal}}-I_{\text{reference}}=({ɛ}_{\text{signal}}-{ɛ}_{\text{reference}})\;\sigma_{e}T^{4}$$

**Figure 4.**
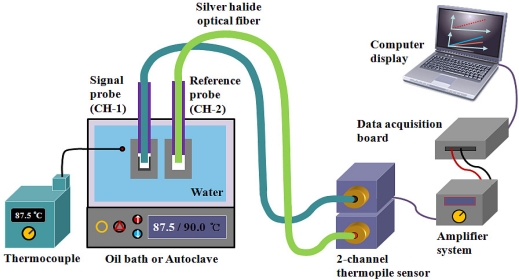
Experimental setup for measuring the temperature of the water using the embedded IR fiber-optic sensor.

Figure 4 shows the experimental setup employed for temperature measurements using the embedded IR fiber-optic sensor. The signal and reference probes and a K-type thermocouple (Fluke, 54II thermometer) were placed in an oil bath (Samheung Energy, SH-OILWB10) with a temperature uniformity of ± 0.5 °C and an autoclave of a high temperature/pressure test loop (UTO Engineering, HT/HP Loop System), which is a secondary system simulation of a nuclear power plant.

**Figure 5.**
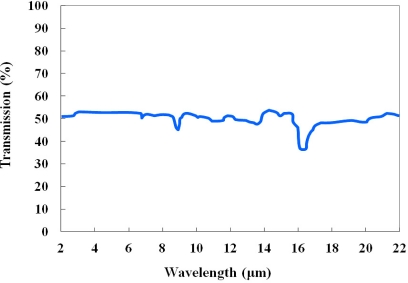
Transmission rates of an IR filter on the thermopile sensor.

Each probe is connected to a 2-channel thermopile-amplifier system, which is composed of two identical thermopiles (Perkin Elmer, A2TPMI334OAA060) and an amplifier system. The thermopile can measure IR radiation at room temperature without cooling, and its sensing range is from 2 to 22 μm, as shown in Figure 5. This sensor has ±1.5 K accuracy at the calibration point when the ambient temperature is 25 ± 1 °C, and the emissivity and the surface temperature of heat source are about 99% and 60 ± 0.3 °C, respectively.

The IR radiation emitted from the two temperature sensing probes according to the temperature variation of the water is guided by the silver halide optical fibers to the 2-channel thermopile sensor. The IR signals are converted to electric signals by the thermopile sensor, and they are then pre-amplified by a programmable chopper-amplifier system. The output signals from the thermopile-amplifier system are transmitted to a data acquisition board (National Instruments, NI USB-6259) and a laptop running LabVIEW (National Instruments). The temperature of the water is controlled and monitored with the temperature controllers in the oil bath or the autoclave. The temperature of the water is determined by measuring the intensity difference between the IR radiation emitted from the two types of IR emitting materials in the caps of the signal and the reference probes. We measured these differences through repeated and random experiments.

## 3. Results

**Figure 6.**
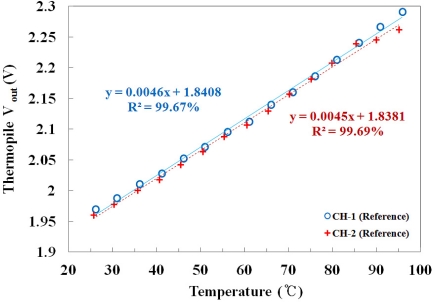
Response measurements of the 2-channel thermopile sensor using the same reference probes for calibration.

To calibrate the thermopile, each channel response of the 2-channel thermopile sensor was measured using the same reference probes in the oil bath. Figure 6 shows the output voltages of two channel thermopiles *versus* the temperature of the water, which was measured using a thermocouple as a function of the IR signal. It can be seen that there is a linear dependence between the IR signal and the water temperature, and the mathematical forms of the best fit lines are also presented in Figure 6. Although the gains and the offset voltages of each channel were similar, the two channels were corrected to be equal with a calibration procedure based on the LabVIEW program to measure the temperature accurately.

**Figure 7.**
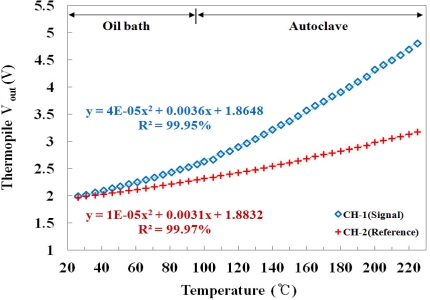
Response of each channel of the embedded IR fiber-optic temperature sensor according to the temperature variation of the water.

Figure 7 shows the output voltages of the embedded IR fiber-optic temperature sensor using the signal and the reference probes according to the temperature variation of the water in the oil bath and the autoclave. The responses of the two channels are dependent on the IR emissivity of each probe. The gradient of CH-1 is steeper than that of CH-2, and the output voltages of CH-1 are higher than those of CH-2 because the emissivity of the signal probe is higher than that of the reference probe. Generally, in radiation thermometry is not easy to measure the temperature for materials which have a low emissivity since they do not emit enough IR radiation, as shown in Figure 7. However, these materials can be used to provide a reference signal because the IR radiations emitted from them do not change much with temperature variations in comparison with those of the materials having high emissivity.

**Figure 8.**
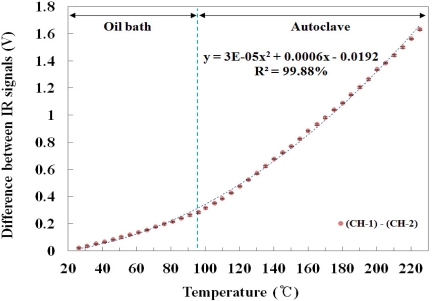
Relationship between the temperature of the water and the difference in IR signals between CH-1 and CH-2.

Figure 8 describes the relationship between the temperature of the water and the difference in the IR signal between the two channels. The difference between two IR signals increased as the temperature of the water increased because the difference in the output voltage between CH-1 and CH-2 gradually increased according to the temperature of the water. The mathematical form of the best fit line to the curve is also presented in Figure 8. In particular, Figures 7 and 8 show that the embedded IR fiber-optic sensor is able to monitor the contact temperature and withstand in conditions of a high temperature/pressure and water-chemistry environment.

**Figure 9.**
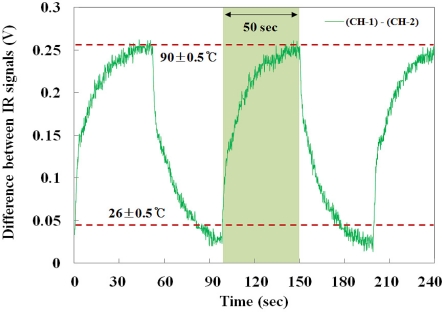
Response time and reproducibility of the embedded IR fiber-optic sensor.

Figure 9 shows the real-time monitoring of the embedded IR fiber-optic temperature sensor to measure response time and reproducibility. In this test, a response time of less than 50 second was measured at a temperature range between 26 ± 0.5 and 90 ± 0.5 °C and the sensing time per degree Celsius could be calculated as 0.78 s/°C. In addition, the proposed temperature sensor also has good reversibility and reproducibility with a percentage standard deviation of 0.587% at 90 ± 0.5 °C, as shown in the figure.

## 4. Conclusions

We have developed an embedded IR fiber-optic temperature sensor using two identical silver halide optical fibers. Induced IR radiation emitted from two temperature sensing probes was measured using a 2-channel thermopile-amplifier system. The relationship between the temperature of the water and the difference in IR signals was also determined. The difference in the amounts of IR signals can be expressed as a function of the temperature of two probes. Therefore, temperature can be measured by monitoring the difference between the IR signals of the two temperature sensing probes.

In this study, we demonstrate that temperature can be determined according to the difference between the amounts of emitted IR radiation from the caps of individual probes. This approach makes it possible to circumvent the emissivity effect of the measured surface of the heat source. Especially, the temperature sensing probe with silver halide optical fibers could withstand a high temperature/pressure and water-chemistry environment. Therefore, thermometry with the proposed sensor can have a high signal-to-noise ratio (SNR), and is immune to variation of parameters such as offset voltage, ambient temperature, and the emissivity and physical conditions of any warm object. Based on the results of this study, it is expected that a 2-channel IR fiber-optic temperature sensor can be developed to accurately monitor temperature in harsh environments.
